# Efficacy of axillary dead space closure after mastectomy, axillary clearance and prosthetic reconstruction: a single-center preliminary experience

**DOI:** 10.3389/fsurg.2024.1401699

**Published:** 2024-07-12

**Authors:** Andrea Lisa, Giulia Bozzo, Valeriano Vinci, Francesco Maria Klinger, Valentina Errico, Corrado Tinterri, Marco Ettore Attilio Klinger, Alberto Testori

**Affiliations:** ^1^ Department of Plastic and Reconstructive Surgery, European Institute of Oncology, IRCCS, Milan, Italy; ^2^ Department of Biomedical Sciences, Humanitas University, Milan, Italy; ^3^ PhD Program in Applied Medical-Surgical Sciences—Department of Surgical Sciences, University of Rome “Tor Vergata”, Rome, Italy; ^4^ Plastic Surgery Unit, IRCCS Humanitas Research Hospital, Milan, Italy; ^5^ Department of Medical Biotechnology and Translational Medicine BIOMETRA, Reconstructive and Aesthetic Plastic Surgery School, University of Milan, Milan, Italy; ^6^ Cancer Center, Division of Thoracic Surgery, IRCCS Humanitas Research Hospital, Milan, Italy; ^7^ Breast Unit, IRCCS Humanitas Research Hospital, Milan, Italy

**Keywords:** breast reconstruction, serome, breast cancer, axillary quilting, reconstruction failure

## Abstract

**Background:**

Postoperative seroma is most frequent after mastectomy (ME) in combination with axillary lymph node dissection (ALND), and its reported incidence varies from 15.5% up to 90%. Seromas can be responsible for discomfort, infections and can lead to reconstruction failure. Therefore, many ways of seroma prevention have been studied, although from a recent overview it has become clear that no single method is reliably successful. Mechanical closure of the dead space, however, was consistently found to be significantly effective. The aim of our study is to evaluate if quilting of the axilla, in patients undergoing ME, immediate prosthetic breast reconstruction and ALND reduces the duration of drain maintenance, the incidence of seromas that require aspiration (clinically significant seromas, CSS) and reconstruction failure rate.

**Materials and methods:**

In our study population we analyzed a total of 81 patients divided into two groups: 27 consecutive patients undergoing mastectomy, axillary lymph node dissection (ALND), breast reconstruction and quilting of the axilla. We subsequently randomly picked up a double number of patients (54) undergoing the same oncological and reconstructive procedures without undergoing axillary quilting, matched for clinical characteristics in order to analyze efficacy of the procedure while reducing any bias. Our observational retrospective data was collected from October 2016 to July 2020 in one single high-volume center. Our median follow-up time was of 40.6 months.

**Results:**

In the case group we observed a reduced time of drain maintenance: 16 vs. 20 days observed in the non-quilted group (*p* < 0.05). Incidence of seromas that required aspiration was 11% in the control group, while 3,7% in the case group. In addition to that, we observed 6 cases of implant removal in the control group, while in the quilted group we only observed a single case.

**Conclusion:**

Previous literature and our results confirm that quilting of the axilla with flap fixation significantly decreases time of drain maintenance, allowing the earlier removal of the drains as well as decreasing the incidence of seroma, its eventual associated complications and related social costs. Moreover, our work suggests how quilting sutures decrease the incidence of seroma in patients undergoing immediate reconstruction, probably reducing the risk for implant removal.

## Introduction

A seroma is a fluid collection, consisting of lymph and serous fluid, which often collects beneath the skin flaps after any form of surgery in the postoperative period. It is the most frequent complication after mastectomy (1), in which case fluid can potentially collect in the breast area or in the dead space of the axilla following sentinel node biopsy (SNB) or axillary node clearance.

Postoperative seroma is most frequent after mastectomy (ME) combined with axillary lymph node dissection (ALND), and its reported incidence varies from 15.5% up to 90% (2–4) in scientific literature.

Seromas themselves can be responsible for various degrees of discomfort and frequent office visits. Interventional procedures aimed to reduce seromas can represent themselves an entry point for microorganisms, therefore being an important risk factor for the development of surgical site infections, which can cause delay of adjuvant therapies or can, in some cases, lead to the necessity of implant removal as an extreme consequence. Moreover, a persistent seroma can necessitate operative management for evacuation, thus a new surgical procedure (5, 6).

Due to its relevant impact, extensive research has been conducted on the pathophysiology of seroma formation. Seroma occurrence has been linked to the extent of breast and axillary lymph node oncologic surgery, type of breast reconstruction as well as the utilization of electrocautery, patients’ comorbidities, and patients’ activity in the postoperative period (4).

In time, different options for seroma prevention have been evaluated and trialed, although from a recent overview it became evident that there is not one single method that taken alone is undoubtedly and clearly successful. Mechanical closure of dead space, however, was the only option that was consistently found to be reliable (5, 7, 8).

However, the effect of quilting the axilla after mastectomy and breast reconstruction has never been evaluated so we decided to perform a clinical retrospective study to assess the effect of dead space mechanical closure in patients who underwent immediate prosthetic breast reconstruction after mastectomy and axillary lymph node dissection (ALND).

## Materials and methods

### Data collection

The studies involving human participants were reviewed and approved by Humanitas Research Hospital ethical committee. Written informed consent to participate in this study was provided by the patient.

Within our study cohort, we examined 81 patients categorized into two groups. Our case group (group A) comprised 27 consecutive patients undergoing conservative mastectomy (skin-sparing, nipple-sparing or skin-reducing), ALND, and breast reconstruction with axillary quilting. The control group (group B) included 54 randomly selected patients undergoing the same oncological and reconstructive procedures but without quilting, balanced in clinical characteristics in order to analyze the effectiveness of the procedure and minimize bias. Clinical characteristics were: age, BMI, expander/prothesis volume, previous radiotherapy (RTP) (Table 1).

**Table 1:** Comparison of clinical characteristics in case and control group.

** **	Group A (27)	Group B (54)	*p*-value
Age (Median, IQR)	47 (37–71)	50 (33–77)	0.65
BMI (Median, IQR)	24.44 (17.24–34.24)	19.66 (19.13–31.64)	0.07
Expander/prosthesis volume, no (%)			
≤400 cc	8 (29.7)	9 (16.6)	0.17
>400 cc	19 (70.3)	45 (83.4)	
Previous radiotherapy, no (%)			
Yes	1 (3.7)	0	0.15
No	26 (96.3)	54 (100)	
Levels of axillary dissection no (%)			
1 + 2	22 (81.5)	46 (85.2)	
3:	5 (18.9)	8 (14.8)	0.91

Cases contributed by each surgeon in both the non-quilted and quilted groups were equated to mitigate bias associated with surgeon techniques or preferences.

Our observational retrospective data was then collected from October 2016 to July 2020 in one single high-volume center. Our mean follow-up time was of 40.6 months.

The amount and color of drained fluid were recorded daily. The drains were removed when the amount of collected fluid became less than 50 cc per day. The duration of drains’ maintenance was recorded for each patient and the results were compared between the two groups.

Pain at rest after breast surgery measured by using a 100 mm VAS scale (0: no pain to 100: severe pain) on postoperative days 1, 2 and 7.

### Surgical technique

All patients received preoperative prophylactic antibiotic therapy (cefazolin 2 g) 30 min before incision, whereas allergic patients received 600 mg of clindamycin.

Mastectomy was performed with electrocautery, making sure to leave adequately thick but oncologically safe mastectomy flaps and preserving pectoralis major muscle and its fascia. ALND was performed by two breast surgeons (the same operators performed every procedure, which reduced any possible bias), I and II lymph node levels were removed and lymph channels clipped (if possible) or bovied. Level III nodes were removed only when hard lymph nodes were palpable between pectoralis major and minor muscles (Table 1). All patients underwent retropectoral breast reconstruction with comparable prosthesis/expander volumes between the two groups (see Table 1). A 19 French drain was inserted in the breast submuscular pocket just before the end of surgery. In case of large breasts (breast weight >400 g) we put a 15 French drain in the subcutaneous breast pocket as well.

In control patients, axillary cavity was not isolated. We adopted the traditional procedure in which the skin flaps are brought together and sutured with a single layer of absorbable 3/0 monofilament interrupted sutures (followed by a subcuticular 4/0 suture), after putting a 19 French drain in the axillary pocket.

On the contrary, in case patients, we quilted the axillary space using a continuous Vicryl 0 suture. More specifically, we sutured the superficial fascia of the axillary region to the pectoralis minor in order to separate the axilla from the thorax and have two different areas of suction, breast pocket and axilla (Supplementary Material Video S1). This process allowed us to reduce the dead space that would favor fluid accumulation. We took care not to create any skin dimpling. Before quilting we inserted a 19 French drain in the axillary pocket. In case of large breasts (breast weight >400 g) we put a 15 French drain in the subcutaneous breast pocket as well.

### Endpoints

The aim of the study is to evaluate if quilting of the axilla, in patients undergoing mastectomy, immediate prosthetic breast reconstruction and ALND reduces the duration of drain maintenance, the incidence of seroma of the breast or the axillary cavity that require aspiration (clinically significant seromas, CSS) and reconstruction failure rate. Clinically significant seroma is thus defined by aspiration and not defined by symptoms or by use of ultrasound (6).

Reconstruction failure is defined by the ultimate necessity to remove the breast implant without performing any other reconstruction due to the impossibility to eradicate the infection.

### Statistics

Data was retrospectively collected and recorded in Microsoft Excel (Microsoft Cor., Redmond, Wash.) on an “*ad hoc*” spreadsheet where clinical data have been reported.

Statistics were performed using SPSS version 19 (IBM Corporation, Armonk, NY, USA). Discrete variables were described as number and percentage or median and interquartile range (IQR), which reports the range between the 25th and 75th percentile; continuous variables as mean and standard deviation. Chi-squared and Fisher's exact tests were utilized as appropriate, and a *p*-value of 0.05 or less was considered statistically significant.

We performed the Shapiro-Wilk test to assess the normality of the data. The *p*-values obtained were 0.87 for all tested variables (age, BMI, duration of drain maintenance). This indicates that we cannot reject the null hypothesis of normality for any of the variables. Therefore, we can assume that the data follow a normal distribution.

## Results

We collected a total of 81 patients, divided into two groups: a case group (A) including 27 patients undergoing quilting of the axilla and a control group (B) made of 54 patients, who underwent traditional surgical procedures. In order to reduce bias we randomly picked control patients having comparable characteristics and known risk factors for seroma development as shown in Table 2.

**Table 2:** Comparison of clinical outcomes in case and control group.

Seroma, no (%)	1 (3.7)	6 (11)	0.26
Duration of drain maintenance (Mean, SD)	16 (5.33)	20 (6.24)	0.01
Failure of reconstruction, no (%)	1 (3.7)	6 (11)	0.26

**Figure 1:**
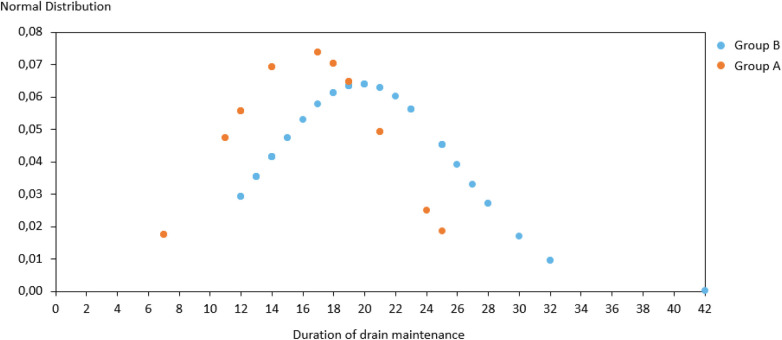
Comparison of mean and standard deviation of drain maintenance duration in control groups.

As previously explained, the amount and color of drained fluid were recorded daily. In the case group we observed a reduced time of drain maintenance: 16 vs. 20 days observed in the non-quilted group (*p* < 0.05) as showed in Figure 1. Color of fluid was always clear and did not change between the two groups.

Incidence of seromas that required aspiration was 11% in the control group, while 3,7% in the case group.

In addition to that, we observed 6 cases of implant removal in the control group, due to infections that developed between day 18 and 19, while in the quilted group we reported only a single case (see Table 2).

Regarding postoperative pain evaluation, we found that there was no significant difference in VAS scores between the 2 groups at day 1, 2 and 7 h after surgery (*P *> 0.05, Table 3).

**Table 3:** Comparison of VAS score between the two groups.

** **	Case	Controls	*P*-value
Day 1	2.4 ± 0.6	2.3 ± 0.6	*P* > 0.05
Day 2	2.6 ± 0.5	2.5 ± 0.6	*P* > 0.05
Day 7	1.2 ± 0.8	1.1 ± 0.9	*P* > 0.05

## Discussion

Every year, around 53,000 women receive a breast cancer diagnosis in Italy and 2.1 million in the world (9–11). Approximately 40% of these patients undergo mastectomy, 30% require ALND, approximately half combined with a mastectomy, with this last group of patients having the highest probability of developing a seroma, which is around 90%. The resulting charge on patients and outpatient clinics is immense, with important consequences on the rise of health budget. In fact, seroma development represents a very important risk factor for a bad reconstructive outcome and reconstruction failure, which is why we decided to introduce the above-mentioned technique in our clinical practice.

There are many methods that can in some way decrease the incidence of seroma, although no single method is truly efficient. Inconsistent results have been reported for shoulder immobilization, pressure dressing and high vs. low drainage systems. Other interventions have been proven ineffective, such as fibrin sealant, bovine thrombin and steroids (6). None of these is in any way as powerful as suturing skin flaps to the underlying muscle as shown in various prospective, randomized controlled trials (12).

Reducing seroma formation is extremely important, especially when immediate breast reconstruction is performed, since fluid accumulation can increase the risk of local infection and eventually of implant removal.

For this reason, we decided to perform a retrospective study to evaluate if quilting of the axillary space after mastectomy, ALND and immediate breast reconstruction could reduce seroma formation and reconstruction failure related.

In order to evaluate our results and reduce bias we compared our study population with a randomly chosen control group of patients (picked from hospital database) having comparable clinical characteristics which are known risk factors for seroma development.

In our case series we observed a reduced time of drain maintenance and rate of seroma aspiration, together with a reduced implant removal rate in the quilted group, although the last two findings were not statistically significant.

As far as we know our analysis is one of the few to investigate mastectomy flap fixation (6, 13) with a higher number of patients included than in some previous works (13).

Moreover in our work, differently from other studies like Gong’s but also ten Wolde’s (6, 12) in which suture of lateral thoracic wall to the pectoralis major muscle was performed, we sutured the thoracic lateral wall subcutaneous tissue to the subcutaneous tissue of the axillary region, which in our opinion could be responsible for the reduced postoperative pain and possibly less bleeding.

Most importantly, in our case series we included patients who received immediate breast reconstruction, which was an exclusion criterion in all previous works. For example in ten Wolde et al's work 176 patients were included, but none of them underwent immediate breast reconstruction (6), in Sakkary's study only 40 patients were included and breast reconstruction was not performed in any of them. In our opinion analyzing clinical efficacy of quilting sutures in patients undergoing immediate breast reconstruction is extremely relevant since development of a seroma has more serious complications than in non-reconstructed patients: it can in fact compromise a reconstruction's outcome and candidate the patient to a new surgery necessary to remove the implant. Indeed, in our case series we observed a critical drop in the total number of implants removed.

Moreover, we observed a reduced incidence of seromas requiring aspiration. In ten Wolde et al.'s study (6), aspirations were necessary in around 91,9% of non-quilted patients and 30,4% of quilted ones (in the subgroup of patients undergoing mastectomy combined with ALND). We believe that one possible reason for the higher rate of aspirations in the cited study could be that immediate reconstruction may reduce the incidence of postoperative seromas, even if this has been reported in just one previous study (2). Another possible explanation is that in his experience drains were removed after 36 h in any case, while we prefer to remove them only when the collected fluid becomes less than 50 cc per day within 21 days in order to reduce implant infections rate (3, 13).

Before introducing such change we rarely managed to fit in this time limit since mean fluid production was higher than 50 cc per day. Conversely, quilting sutures, by reducing fluid production, allowed a faster drain removal, 16 days as a mean value, which could explain the reduced reconstruction failure rate related to infections.

The results described seem promising, however, quilting could have some potential disadvantages. In particular, quilting sutures could be responsible for extra pain associated to mastectomy and ALND so that more analgesics in the postoperative period might be required. In addition to that, quilting the axilla could be responsible for reduced shoulder mobility and subsequent hypothetical long-term effects such as chronic pain.

However, in our experience we did not observe a worse pain control or altered mobility in patients undergoing quilting of the axilla.

On the other hand, suturing the subcutaneous tissue could determine skin-dimpling, and particular care should be used to reduce its occurrence. In our experience we observed 4 patients (among the first patients in whom we adopted quilting sutures) who developed some degree of skin-dimpling that was solved with massage therapy in the long-term.

We are aware our work has some limitations: our analysis is a single institution retrospective study and has a relatively small number of patients included. We believe that a wider sample with a prospective design could give a stronger evidence and guarantee more attendable results. In addition to that, we aim to include in future analysis an assessment of postoperative quality of life, including pain and shoulder mobility as well.

## Conclusion

Previous literature and our results confirm that quilting of the axilla with flap fixation significantly decreases time of drains maintenance, allowing earlier removal of drains as well as decreasing the incidence of seroma, its eventual associated complications and related social costs. Moreover, our work suggests how quilting sutures, decreasing the incidence of seroma in patients undergoing immediate reconstruction, probably reduces the risk for implant removal.

Although further evidence is needed, we believe such a procedure can be useful and should be introduced as a step in breast surgery in order to reduce overall complications (13, 14).

## Data Availability

The raw data supporting the conclusions of this article will be made available by the authors, without undue reservation.

## References

[1] Kuroi K Shimozuma K Taguchi K Imai H Yamashiro H Oshumi S Pathophysiology of seroma in breast cancer. Breast Cancer. (2005) 12(4):288–93. 10.2325/jbcs.12.288 16286909

[2] Woodworth PA McBoyle MF Helmer SD Beamer RL. Seroma formation after breast cancer surgery: incidence and predicting factors. Am Surg. (2000) 66(5):444–50. discussion 450-1. 10.1177/000313480006600505 10824744

[3] Tadych K Donegan WL. Postmastectomy seromas and wound drainage. Surg Gynecol Obstet. (1987) 165(6):483–7. PMID: 3686312 3686312

[4] Nadkarni MS Rangole AK Sharma RK Hawaldar RV Parmar VV Badwe AR. Influence of surgical technique on axillary seroma formation: a randomized study. ANZ J Surg. (2007) 77(5):385–9. 10.1111/j.1445-2197.2007.04067.x 17497983

[5] Stanczyk M Grala B Zwierowicz T Maruszynki M. Surgical resection for persistent seroma, following modified radical mastectomy. World J Surg Oncol. (2007) 5:104. 10.1186/1477-7819-5-104 17888182 PMC2082032

[6] ten Wolde B van den Wildenberg FJH Keemers-Gels ME Polat F Strobbe LJA. Quilting prevents seroma formation following breast cancer surgery: closing the dead space by quilting prevents seroma following axillary lymph node dissection and mastectomy. Ann Surg Oncol. (2014) 21(3):802–7. 10.1245/s10434-013-3359-x 24217790

[7] van Bemmel AJM van de Velde CJH Schmitz RF Liefers GJ. Prevention of seroma formation after axillary dissection in breast cancer: a systematic review. Eur J Surg Oncol. (2011) 37(10):829–35. 10.1016/j.ejso.2011.04.012 21849243

[8] Degnim AC Hoskin TL Brahmbhatt RD Warren-Peled A Loprinzi M Pavey ES Randomized trial of drain antisepsis after mastectomy and immediate prosthetic breast reconstruction. Ann Surg Oncol. (2014) 21(10):3240–8. 10.1245/s10434-014-3918-9 25096386 PMC4373621

[9] Barni S Venturini M Molino A Donadio M Rizzoli S Maiello E Importance of adherence to guidelines in breast cancer clinical practice. The Italian experience (AIOM). Tumori. (2011) 97(5):559–63. 10.1177/030089161109700503 22158483

[10] Harbeck N Penault-Llorca F Cortes J Gnant M Houssami N Poortmans P Breast cancer. Nat Rev Dis Primers. (2019) 5(1):66. 10.1038/s41572-019-0111-2 31548545

[11] Bray F Ferlay J Soerjomataram I Siegel RL Torre LA Jemal A. Global cancer statistics 2018: GLOBOCAN estimates of incidence and mortality worldwide for 36 cancers in 185 countries. CA Cancer J Clin. (2018) 68(6):394–424. 10.3322/caac.21492 30207593

[12] Gong Y Xu J Shao J Cheng H Wu X Zhao D Prevention of seroma formation after mastectomy and axillary dissection by lymph vessel ligation and dead space closure: a randomized trial. Am J Surg. (2010) 200(3):352–6. 10.1016/j.amjsurg.2009.10.013 20409509

[13] Sakkary MA. The value of mastectomy flap fixation in reducing fluid drainage and seroma formation in breast cancer patients. World J Surg Oncol. (2012) 10:8. 10.1186/1477-7819-10-8 22236813 PMC3279306

[14] Chilson TR Chan FD Lonser RR Wu TM Aitken DR. Seroma prevention after modified radical mastectomy. Am Surg. (1992) 58(12):750–4. PMID: 1456600

